# Supplementary material for: Causal interplay between lactose intolerance and gut microbiota: a combined bidirectional Mendelian randomization and in vivo validation study
Source: Front Nutr. 2026 Jun 1;13:1803337. doi: 10.3389/fnut.2026.1803337 (PMC13265576; doi:10.3389/fnut.2026.1803337)
Supplement: Supplementary file 1 [file Data_Sheet_1.zip › supplementary materials/Forward/leave one out cross validation/ebi-a-GCST90027834.finngen_R12_E4_LACTONAS.pdf]

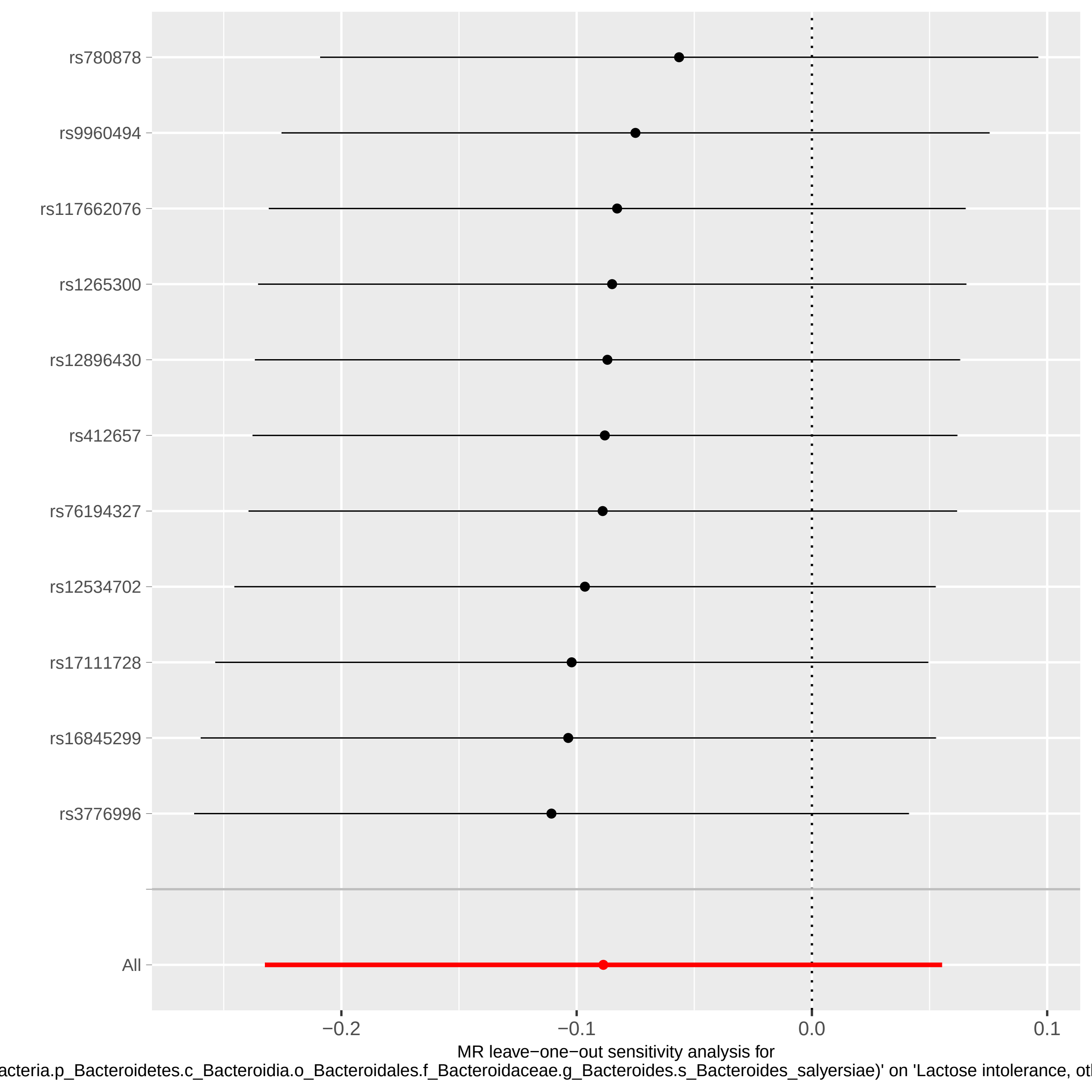
rs780878
rs9960494
rs117662076
rs1265300
rs12896430
rs412657
rs76194327
rs12534702
rs17111728
rs16845299
rs3776996
All
−0.2
−0.1
0.0
0.1
MR leave−one−out sensitivity analysis for
acteria.p_Bacteroidetes.c_Bacteroidia.o_Bacteroidales.f_Bacteroidaceae.g_Bacteroides.s_Bacteroides_salyersiae)' on 'Lactose intolerance, ot
